# The Level of Health Literacy of Seniors Living in Eastern Region of Poland. Preliminary Study

**DOI:** 10.3390/healthcare8030277

**Published:** 2020-08-17

**Authors:** Bogumiła Kosicka, Alina Deluga, Jadwiga Bąk, Justyna Chałdaś-Majdańska, Monika Bieniak, Michał Machul, Agnieszka Chrzan-Rodak, Krzysztof Jurek, Beata Dobrowolska

**Affiliations:** 1 Department of Management in Nursing, Faculty of Health Sciences, Medical University of Lublin, 20-081 Lublin, Poland; bogumila.kosicka@umlub.pl; 2 Department of Family Medicine and Community Nursing, Faculty of Health Sciences, Medical University of Lublin, 20-081 Lublin, Poland; alina.deluga@umlub.pl (A.D.); agnieszkachrzan607@gmail.com (A.C.-R.); 3 Department of Paediatric Nursing, Faculty of Health Sciences, Medical University of Lublin, 20-081 Lublin, Poland; jadwigabak25@gmail.com; 4 Department of Development in Nursing, Faculty of Health Sciences, Medical University of Lublin, 20-081 Lublin, Poland; justynachaldas@wp.pl (J.C.-M.); monika675@poczta.onet.pl (M.B.); mm.machul@gmail.com (M.M.); 5 Faculty of Social Sciences, Institute of Sociology, John Paul II Catholic University, 20-950 Lublin, Poland; kjurek@interia.eu

**Keywords:** elderly, health literacy, HLS-EU 47 tool, eastern Poland

## Abstract

Health literacy (HL) is recognised as an important, modifiable factor in the self-management and health performance of elderly people. The aim of this preliminary study was to identify and analyse the level of health literacy among the elderly living in one of the eastern regions in Poland. The cross-sectional study was conducted among a convenience sample of 200 seniors aged 65+ after cognitive pre-screening with the use of the Montreal Cognitive Assessment (MoCA) scale. To collect data, the Polish version of the HLS-EU-Q47 was used. More than half of the elderly surveyed presented problematic levels of general HL (GEN-HL), and also problematic levels of other dimensions: health care health literacy (HC-HL), disease prevention health literacy (DP-HL), and health promotion health literacy (HP-HL). The level of seniors’ HL is dependent on the level of their education, place of living, participation in activities run by Daily Center for the Elderly, and their self-assessment of health condition (*p* < 0.05). These results imply the important message that there is a need to create initiatives and programs improving health literacy targeted at seniors living in rural areas, those with lower levels of education, and those with poor access to activities organised by institutions supporting seniors.

## 1. Introduction

Health literacy (HL) is an evolving concept that has been gaining in importance over the last decade [1]. According to the definition proposed by Sørensen in 2012: “Health literacy is linked to literacy and entails people’s knowledge, motivation, and competences to access, understand, appraise, and apply health information in order to make judgements and take decisions in everyday life concerning healthcare, disease prevention, and health promotion to maintain or improve quality of life during the life course” [2].

HL is recognised as an important determinant of society’s health and, thus, a key factor in efforts to improve it [3,4]. Many studies have shown that low levels of HL are of prognostic importance for various negative health effects [2,5,6,7]. It is also related to higher health care costs [8,9]. Research shows that a lower degree of health knowledge is associated with poorer mental and physical health [8] and adverse health behaviours such as: lower ability to take care of oneself [10], higher percentage of avoidable hospitalisations [6,11], and less frequent use of preventive health services [12,13]. People with low levels of HL are less likely to benefit from screening, and have considerable difficulty in understanding their disease, their treatment plan, and especially the treatment of chronic diseases [14,15,16,17]. Low HL is also associated with higher mortality and lower levels of care satisfaction [1,9].

HL is often referred to as an important indicator in population health monitoring [16]. Based on HL surveys in eight European countries, around 50% of European adults have low levels of health literacy [18]. These people have serious problems with health-related tasks and situations [19,20]. The elderly has been indicated as a group particularly vulnerable to health literacy deficits [20,21,22].

In an era of rapid medical and technological progress and changes in the way health care is provided, society requires high levels of health literacy [20,23]. Changes in the health systems of many countries pose increasingly greater requirements of society to take greater responsibility for its health [7,24]. Nevertheless, there is still a growing gap between the demand for health literacy and the actual health literacy of many patients [18,25]. Many studies have shown that HL levels are closely related to age [21,25,26]. Limited health literacy is more prevalent among older adults [22,27]. Knowledge and understanding of the factors related to the health literacy of the elderly can reduce the health problems of this group in all communities. 

According to official forecasts, the population of people aged 65 and above in the European Union (EU) will increase to 148.3 million in 2060. As regards people aged 80 and over, according to the forecasts, their number will increase to 61.7 million in 2060. Consequently, the share of pensioners in the population will record a sharp increase to 50.1% in 2060 [28]. The low level of health knowledge is a particularly important problem among people over 65 years of age, most of whom achieve results below the basic levels of health literacy [26]. Health literacy is an important, modifiable factor in the self-management and health performance of elderly people [22,29]. Socio-economic status (lower income, poor education), age, level of education (lower education), and changes in cognitive and physical abilities associated with ageing are all factors contributing to lower health literacy among elderly people [30,31]. 

Health literacy is well explored in many different populations around the world, including the elderly population. However, in many studies, the elderly constituted only part of the studied group, e.g., in the Polish national research by Słońska et al. [32] and Duplaga [33]. Therefore, our intention in this current study was to focus only on seniors’ health literacy level, collecting data in face-to-face interviews after cognitive pre-screening of every respondent. The astern part of Poland—Lubelskie Voivodship—was chosen, as it is a region that may serve as a specific lens on which the main problems of an aging population can be focused. Considering the prognosis of the Statistical Office, the Lubelskie Voivodeship will be among the fastest ageing Polish voivodships with a so-called double ageing, in which the highest increase is observed in the number of people aged 80+ [34,35]. Research regarding health literacy in this population may support discussions and initiatives regarding micro, mezzo, and macro strategies to maintain healthy ageing with longer independence of seniors in this age group.

## 2. Materials and Methods 

### 2.1. Aim

The aim of the study was to identify and analyse the level of health literacy among the elderly living in one of the eastern regions in Poland.

### 2.2. Study Participants and Setting

The study was carried out as part of the Erasmus+ project ‘*Healthy lifestyle for aging well (HLAW)’* among convenience sample of 200 seniors aged 65+, living in one of the eastern regions in Poland. The study was carried out in day-care centres for the elderly, senior citizens’ clubs, the University of the Third Age, and the respondents’ living environment. Prepared interviewers met with seniors, explained the purpose and process data collection, asked for the consent to participate in the study, and when consent was given, assisted seniors during filling in the questionnaire in case of any difficulties. The return rate of the surveys was at 69%. A total of 150 questionnaires were obtained, 138 of which were correctly filled in and accepted for analysis.

### 2.3. Research Instruments

The basis for the qualification of seniors for the study was the use of the Montreal Cognitive Assessment (MoCA) scale, which is a screening tool to detect mild cognitive disorders. Behaviour is assessed, including: abstraction, short-term memory, visual-spatial functions, executive functions, language, verbal fluency, allopsychic orientation, and attention [36]. Persons who received at least 26 points on a maximum scale of 30 points were eligible for the further stage of research.

To collect the material, the Polish version of the HLS-EU-Q47 research tool was used, which is a part of the European Health Literacy Survey HLS-EU. The HLS-EU survey was conducted in eight European countries in 2011 [18,37]. Consent for the use of the Polish version of the questionnaire was given by the Polish coordinator of HLS-EU, Zofia Słońska [32].

The HLS-EU-Q47 questionnaire contains 47 questions, which cover the different stages of information processing (finding health information, understanding health information, judging health information, and applying health information) in three main dimensions: health care health literacy (HC-HL), disease prevention health literacy (DP-HL), and health promotion health literacy (HP-HL). Respondents participating in the survey evaluate individual questions according to four categories of answers (very easy, rather easy, rather difficult, very difficult). The HLS-EU-Q47 tool enables us to calculate a general-HL index, comprising all items and providing a general picture and overview of respondents’ health literacy (GEN-HL), and it provides an index for all three dimensions of health literacy: HC-HL, DP-HL, and HP-HL.

The Cronbach alpha coefficient for the Polish version of the scale measured in this current study was as follows: GEN-HL: 0.90, HC-HL: 0.95, DP-HL: 0.94, and HP-HL: 0.94.

### 2.4. Analysis

Quantitative variables were described by mean and standard deviation. To compare the two groups, the Mann–Whitney test was used. Differences among three and more groups were verified by the Kruskal–Wallis test. Statistical analyses were performed using IBM SPSS Statistics 23. Statistically significant results accepted were *p* ≤ 0.05.

According to authors of the HLS-EU-Q47 [18,37], the scores for general HL, health care HL, disease prevention HL, and health promotion HL were categorised into ‘inadequate HL’: score 0–25, ‘problematic HL’: score 25.01–33, ‘sufficient HL’: score 33.01–42, and ‘excellent HL’: score 42.01–50.

### 2.5. Ethical Aspects

The research was conducted in accordance with the research protocol approved by the Ethical Committee at the Medical University of Lublin—no KE-0254/31/2016—and the principles of the Helsinki Declaration. Each of the respondents gave their voluntary consent in writing before the start of the study and were informed about the purpose of the study. Consent was also obtained from the directors of the institutions where research was carried out. 

## 3. Results

### 3.1. Characteristics of the Studied Group

Participants were predominantly female (65.2%). The age of respondents ranged from 65 to 94 years (M = 72.41, SD = 6.90). Most of them had secondary (42.8%) and primary (40.6%) education. More than half of the elders (56.5%) were living in urban areas and with family (39.9%). More than half (55.8%) of the elders rated their personal health as ‘bad’ or ‘fair’. More detailed data are included in Table 1.

### 3.2. The General Level of Health Literacy Among Seniors

Health literacy of seniors is on average somewhat higher for health care (M = 32.82) or disease prevention (M = 31.83) than for health promotion (M = 31.02) (Table 2).

**Figure 1:**
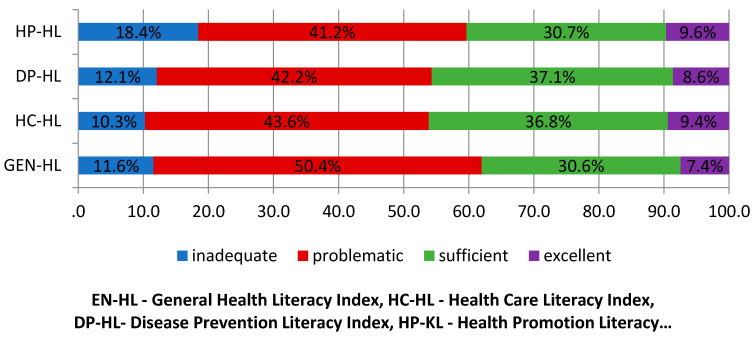
Percentages of HL indices levels thresholds.

More than half of the elderly surveyed presented a problematic level of general HL (50.4%), and also a problematic level of other dimensions of HL (Figure 1).

### 3.3. HLS-EU Health Literacy Indices of Seniors vs Their Sociodemographic Characteristics

No statistical differences between men and women were found regarding GEN-HL and all three dimensions of HL. Seniors with higher education tend to have higher health literacy level in general and in all three dimensions, as compared to seniors with primary and secondary education. Differences for GEN-HL and HC-HL are statistically significant (*p* < 0.05). Seniors from urban area obtained higher mean results for GEN-HL and all three dimensions of HL than seniors from rural areas, and these differences are statistically significant (*p* < 0.05). The age of the seniors did not differentiate their mean results for GEN-HL and all its dimensions. Seniors who participated in activities run by the Daily Center for the Elderly or other organisations/institutions and those who self-assessed their health condition as very good and good obtained higher mean results for GEN-HL and two out of the three dimensions of HL. Differences are statistically significant (*p* < 0.05). (Table 3). 

Seniors aged over 75 years (58.0%), those with higher education (55%), and those who self-assessed their health condition as fair or bad (61.1%) more often presented a problematic level of GEN-HL. Seniors from rural areas (16.3%) and those who did not participate in activities run by the Daily Center for the Elderly or other organisations/institutions (15.9%) more often showed inadequate levels of GEN-HL than seniors from urban areas (8.3%) and seniors who admitted their participation in such activities (2.6%). None of the seniors with higher education presented an inadequate level of GEN-HL (Figures S1–S6 in supplementary materials). 

## 4. Discussion

Improving people’s health literacy is one of the most basic, effective, and economic measures to improve the health of the whole population [9,22]. Improved HL levels contribute to increased health outcomes, well-being, and reduced health inequalities [39].

The aim of this study was to determine the level of health literacy in the group of people above 65 years old. The study showed that 62% of the surveyed people had insufficient General HL, (11.6% inadequate, 50.4% problematic), and only 7.4% had excellent General HL. This is a significantly lower HL than in people under 65 years of age. Research conducted by Słońska et al. [32] indicated that in the group of people over 65 years of age, the percentage of people with insufficient HL was the highest when comparing with younger respondents, and it exceeded 60%. Similarly, in another study that evaluated health literacy according to demographic characteristics of Americans aged 16 and over, the oldest group of adults (65 and over) had the lowest HL levels [26]. In our study, there were no statistical differences between respondents representing early and late elderly groups; however, those over 75 years of age more often presented problematic levels of GEN-HL.

As it is shown by other studies, as people get older, they have limited resources such as cognitive skills and social capital, resulting in limited access to information about health services [40,41,42,43]. Therefore, following the results of this current study, interventions that enhance health literacy should be particularly planned for the ageing population. The main aim is to make older adults more independent when searching for information about health and protection, and in understanding and applying this information in their daily lives for the successful self-management of long-term conditions [44].

This study assesses HL in three health-related areas: health care (HC-HL), disease prevention (DP-HL), and health promotion (HP-HL). In all areas studied, the level of HL was insufficient in more than half of the elderly people. The biggest deficits in HL were found in the area of health promotion (18.4% inadequate and 41.2% problematic). Few studies have been conducted that have mainly focused on the impact of health literacy in relation to health promotion alone [45]. One such study on the relationship between health literacy and health-related behaviour was conducted in the context of health promotion in China. It was concluded that in order to reduce risky habits, educational interventions to improve health awareness should be carried out simultaneously within the framework of health promotion activities [46].

Our current research has shown that factors such as education level, place of residence, and participation in activities run by the Daily Center for the Elderly or other organisations/institutions significantly differentiate the HL level of elderly people. People with higher education, who live in the city, and who participate in activities run by the Daily Center for the Elderly or other organisations/institutions were more likely to have sufficient and/or excellent HL. The results of Cordasco et al. [47] and Patel et al. [48] confirm that education is the strongest predictor of health knowledge and clearly shows the importance of education in shaping health literacy.

Our study has also shown that people who perceive their health as very good or good have a significantly higher level of HL than those who assess their health condition badly. Statistically significant differences have been shown in both the general level of HL and in the area of HC-HL and HP-HL. This result is consistent with studies by other authors. The National Assessment of Adult Literacy Report [26] in the USA indicates that 16% of adults (50 million people) who do not have basic health knowledge report their health as poor (42%) more often than adults with good health knowledge. These people are also less likely to benefit from preventive health measures. They make greater use of services designed to treat complications of the disease. The same people tend to use the health care system only when they are very ill, which consequently increases the length of treatment and reduces positive health outcomes [49].

Other studies have shown that having limited literacy and numeracy also acts as an independent risk factor for ill health, leading to medication errors and insufficient understanding of the disease and treatment [50]. In addition, there is a link between literacy and health outcomes that directly corresponds to several health-related disadvantages, such as health and health care knowledge, hospitalisation, and some chronic diseases. Poor knowledge of health issues and limited access to education can lead to a deficiency in managing one’s own health potential [51].

In the context of the unsatisfactory level of health literacy among the elderly and the growing ageing population of European society, there is a great need to increase the level of knowledge about health among people over 65 years of age. The results of the presented study allow us to consider the elderly as a particularly vulnerable group also in the context of health literacy. Taking into account the increase in the dynamics of the demographic process and the reduction of the caring potential of families, it is recommended that we develop an effective, easily accessible system of care for the elderly, in which health education would play a crucial role. In order to be effective, health education requires tailored communication based on trust developed between health care professionals and seniors as a facilitating factor for meeting their health literacy needs [44]. This, in turn, requires specific skills of health professionals, knowledge of the learning styles of seniors, their cognitive and technical resources, and the time to conduct individualised education.

It is worth stressing that the approach towards health literacy in the field of public health is based on the premise that health literacy is ‘not only a personal resource with leads to personal benefits, e.g., healthier life choices and effective use of available health services’ [3], but also a community source that enables community action on health and allows for better control of social and environmental health determinants.

### Limitations

Our study has several limitations. The main limitation is the relatively small number of seniors who took part in the study and the convenience sampling methodology, which is a non-probabilistic method and, as such, prevents the generalizability of the result. Even though seniors were willing to cooperate and most of them agreed to take part in the study, not all of them fulfilled the criteria of a minimum of 26 points on the MoCa scale to be eligible for the study group. Another limitation is in regards the questionnaire used in the study, which consisted of 47 items. It could had made respondents confused in providing reliable answers to all the questions included in the questionnaire. Additionally, the eligible seniors represented only one of regions from Poland, which can be seen as a selection bias.

## 5. Conclusions

Our study revealed that the level of health literacy of seniors aged 65+ is insufficient. Among variables that differentiate the level of seniors’ health literacy are the level of education, place of living, self-assessment of health condition, and participation in activities run by different institutions that support healthy ageing. These results imply an important message that there is a need to create initiatives and programs improving health literacy targeted at seniors living in rural areas, those with lower levels of education, and those with poor access to activities organised by institutions supporting seniors. The improvement of health literacy may help to improve the control of elders over their health and its determinants, and to maintain their independence in this regard. More research of a qualitative nature would make it possible to deepen knowledge regarding the reasons of poor health literacy among seniors and their expectations in its improvement.

## Figures and Tables

**Table 1:** Demographic and socio-economic variables.

Demographic and Socio-economic Variables (*n* = 138)	*n*	%
**Gender**		
Male	48	34.8
Female	90	65.2
Age *		
Up to *74*	85	61.6
*75* and older	53	38.4
**Education**		
Primary education	56	40.6
Secondary education	59	42.8
Tertiary education	23	16.7
**Place of residence**		
Rural residents	60	43.5
Urban residents	78	56.5
**Household living situation**		
Alone	44	31.9
With spouse or partner	6	4.3
With family	55	39.9
With spouse or partner and with family	33	23.9
**Participated in activities run by Daily Center for the Elderly or other organisations/institutions**		
Yes	44	31.9
No	94	68.1
**Self-assessment of health condition**		
Very good	9	6.5
Good	52	37.7
Fair	68	49.3
Bad	9	6.5

* division of age of respondents in two groups: 65–74: ‘early elderly;’ and over 75: ‘late elderly’ [38].

**Table 2:** Means results in HL Indices and the level of HL.

HL Indices	Descriptive Statistics		The Level of HL							
			Inadequate		Problematic		Sufficient		Excellent	
	M	SD	M	SD	M	SD	M	SD	M	SD
**GEN-HL**	31.70	6.63	20.35	3.99	29.73	2.27	35.76	2.31	46.10	2.44
**HC-HL**	32.82	6.99	20.33	3.62	29.92	1.76	35.54	2.53	46.13	1.91
**DP-HL**	31.83	7.86	17.71	5.49	28.96	2.21	35.52	2.68	44.26	2.27
**HP-HL**	31.02	7.71	20.05	4.07	29.36	1.95	34.78	2.13	47.10	2.28

GEN-HL—General Health Literacy Index, HC-HL—Health Care Literacy Index, DP-HL—Disease Prevention Literacy Index, HP-KL—Health Promotion Literacy Index, M—mean value, SD—standard deviation.

**Table 3:** Mean Scores for GEN-HL, HC-HL, DP-HL, and HP-HL by gender, education, place of residence, age, participation in activities run by Daily Center for the Elderly or other organisations/institutions, and self-assessment of health condition.

**HL Indices**	**Gender**							
	**Male**				**Female**		**Statistic**	
	**M**		**SD**		**M**	**SD**	**Z**	***p***
**GEN-HL**	30.93		7.27		32.10	6.28	−0.690	0.490
**HC-HL**	31.47		7.75		33.43	6.58	−0.900	0.368
**DP-HL**	30.65		8.82		32.47	7.27	−1.037	0.300
**HP-HL**	31.03		7.71		31.01	7.76	−0.015	0.988
	**Level of Education**							
	**Primary Education**		**Secondary Education**		**Tertiary Education**		**H**	***p***
	**M**	**SD**	**M**	**SD**	**M**	**SD**		
**GEN-HL**	29.66	5.94	32.45	7.22	34.34	5.18	**6.211**	**0.045**
**HC-HL**	30.44	6.30	33.10	6.89	36.90	6.79	**10.183**	**0.006**
**DP-HL**	29.32	7.60	33.30	8.27	34.11	5.66	5.319	0.070
**HP-HL**	29.30	6.27	31.32	8.58	34.47	7.47	3.935	0.140
	**Rural Area**				**Urban Area**		**Z**	***p***
	**M**		**SD**		**M**	**SD**		
**GEN-HL**	29.72		6.11		33.06	6.66	**−2.179**	**0.029**
**HC-HL**	30.69		6.74		34.20	6.84	**−2.257**	**0.024**
**DP-HL**	29.56		6.97		33.41	8.11	**−2.265**	**0.024**
**HP-HL**	28.54		6.75		32.89	7.90	**−2.653**	**0.008**
	**Age**							
	**Up to 74**				**75 and Older**		**Z**	***p***
	**M**		**SD**		**M**	**SD**		
**GEN-HL**	32.17		6.91		31.04	6.21	−0.679	0.497
**HC-HL**	33.19		7.36		32.24	6.42	−0.643	0.521
**DP-HL**	32.30		7.87		31.12	7.88	−0.242	0.809
**HP-HL**	31.42		8.26		30.45	6.89	−1.044	0.296
	**Participation in Activities Run by Daily Center for the Elderly or Other Organisations/Institutions**							
	**Yes**				**No**		**Z**	***p***
	**M**		**SD**		**M**	**SD**		
**GEN-HL**	34.25		6.97		30.49	6.14	**−2.133**	**0.033**
**HC-HL**	34.99		7.14		31.72	6.69	−1.845	0.065
**DP-HL**	35.17		7.53		30.19	7.54	**−2.528**	**0.011**
**HP-HL**	34.35		8.78		29.29	6.50	**−2.337**	**0.019**
	**Self-Assessment of Health Condition**							
	**Very Good/Good**				**Fair/Bad**		**Z**	***p***
	**M**		**SD**		**M**	**SD**		
**GEN-HL**	33.17		7.13		30.70	6.11	−2.327	**0.020**
**HC-HL**	34.31		7.47		31.74	6.46	−2.181	**0.029**
**DP-HL**	32.28		8.39		31.49	7.50	−0.947	0.344
**HP-HL**	33.21		8.23		29.42	6.94	−2.568	**0.010**

GEN-HL—General Health Literacy Index, HC-HL—Health Care Literacy Index, DP-HL—Disease Prevention Literacy Index, HP-KL—Health Promotion Literacy Index, M—mean value, SD—standard deviation, Z—Mann–Whitney U, H—Kruskal–Wallis Test.

## Supplementary Materials

The following are available online at https://www.mdpi.com/2227-9032/8/3/277/s1, Figure S1: Percentages of HL Indices Levels Thresholds by gender; Figure S2: Percentages of HL Indices Levels Thresholds by age; Figure S3: Percentages of HL Indices Levels Thresholds by education; Figure S4: Percentages of HL Indices Levels Thresholds by place of living; Figure S5: Percentages of HL Indices Levels Thresholds by participation in activities run by Daily Center for the Elderly or other organizations/institutions; Figure S6: Percentages of HL Indices Levels Thresholds by Self-assessment of health condition
