# Supplementary material for: The Level of Health Literacy of Seniors Living in Eastern Region of Poland. Preliminary Study
Source: Healthcare (Basel). 2020 Aug 17;8(3):277. doi: 10.3390/healthcare8030277 (PMC7551014; doi:10.3390/healthcare8030277)
Supplement: Supplementary file 1 [file healthcare-08-00277-s001.pdf]

Supplementary materials for the study: ***The level of health literacy of seniors as measured by the HLS-EU 47 tool***

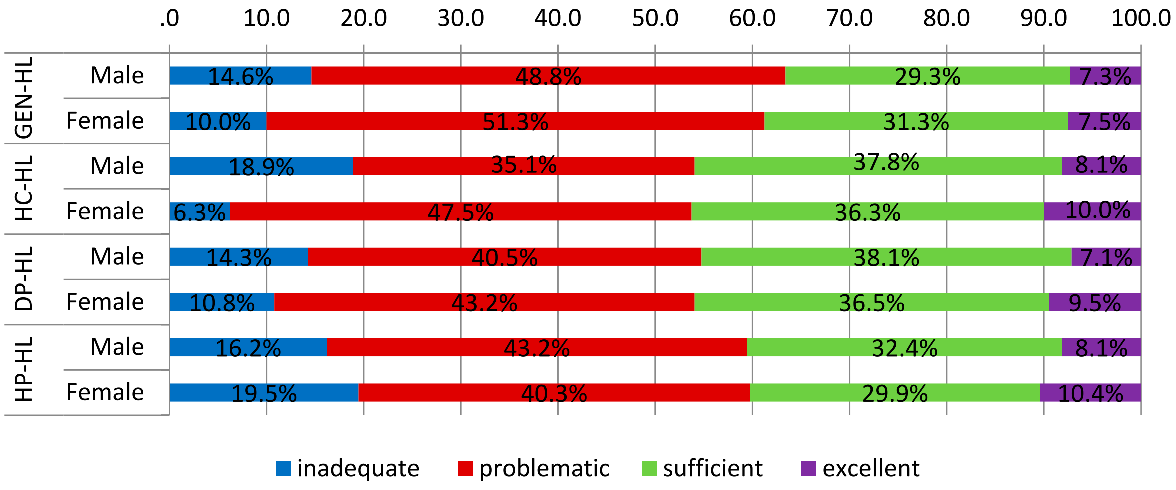

Figure S1. Percentages of HL Indices Levels Thresholds by gender.

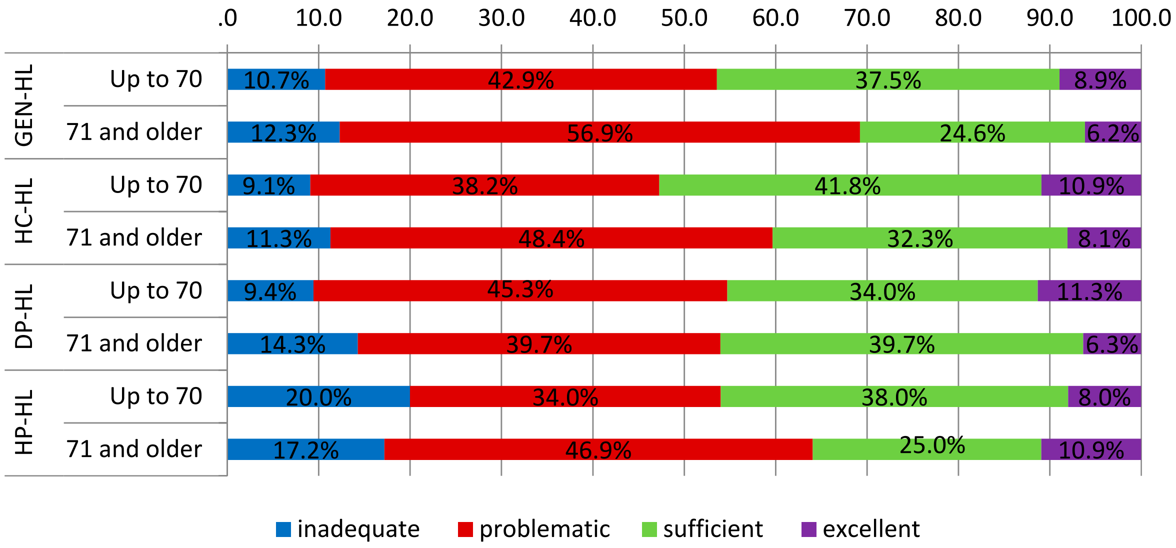

Figure S2. Percentages of HL Indices Levels Thresholds by age.

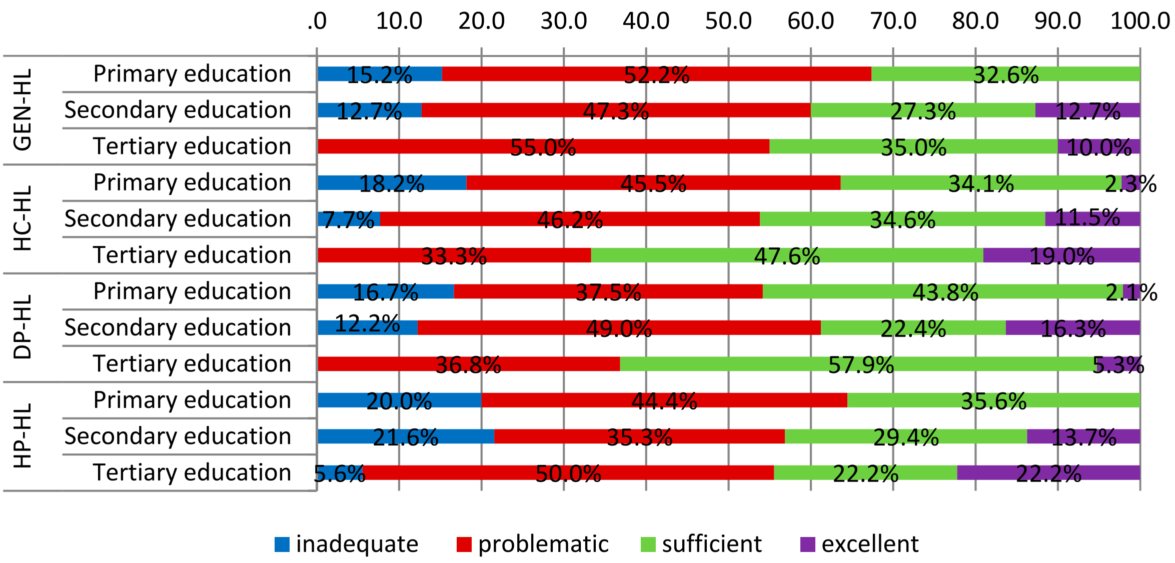

Figure S3. Percentages of HL Indices Levels Thresholds by education.

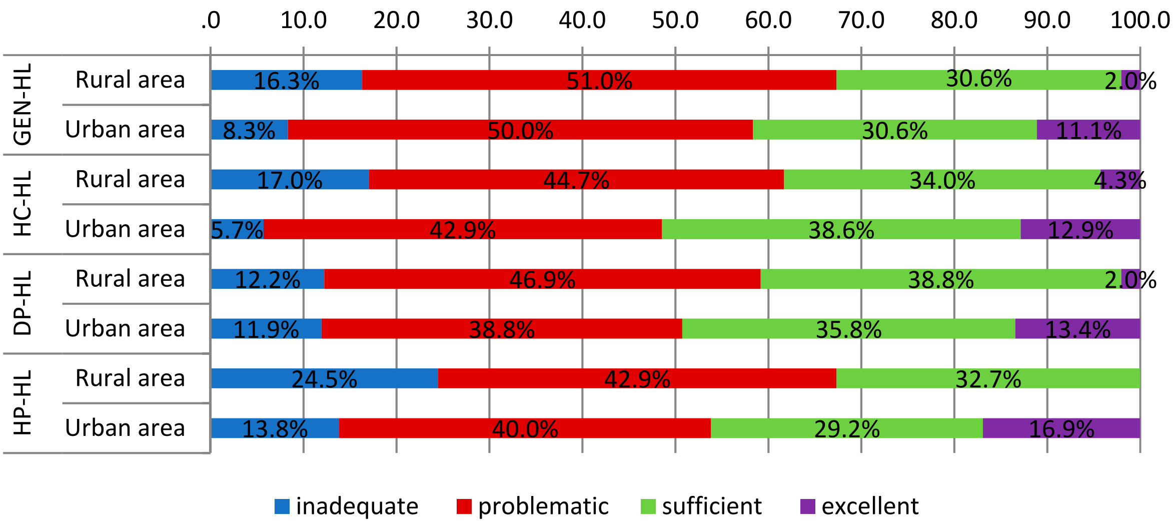

Figure S4. Percentages of HL Indices Levels Thresholds by place of living.

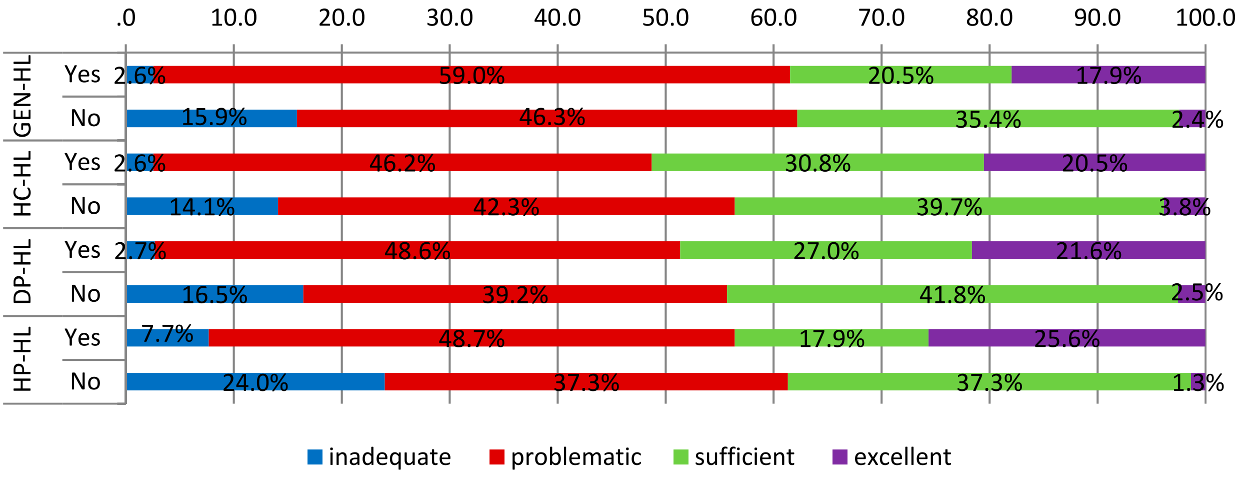

Figure S5. Percentages of HL Indices Levels Thresholds by participation in activities run by Daily Center for the Elderly or other organizations/institutions.

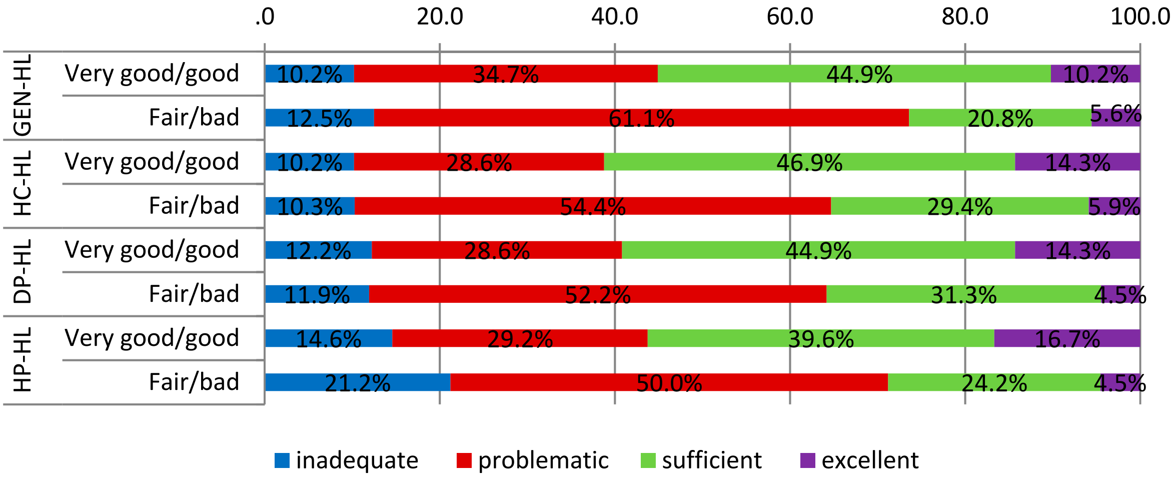

Figure S6. Percentages of HL Indices Levels Thresholds by Self-assessment of health condition.
